# Elastosynth—An Open-Source Software Package for the Generation of Realistic In-Silico RF Data for Ultrasound Elastography

**DOI:** 10.3390/s26216869

**Published:** 2026-10-30

**Authors:** Matthew Caius, Zhenbang Wang, Abbas Samani

**Affiliations:** 1 School of Biomedical Engineering, Western University, London, ON N6A 3K7, Canada; mcaius@uwo.ca; 2 Department of Electrical and Computer Engineering, Western University, London, ON N6A 5B9, Canada; zhenbang.wang@uwo.ca; 3 Departments of Medical Biophysics, Western University, London, ON N6A 5C1, Canada

**Keywords:** ultrasound elastography, *in silico*, FIELD II, radio-frequency data, open source, software package

## Abstract

Ultrasound elastography provides quantitative tissue information, yet displacement algorithm validation is hampered by lack of clinical ground truth and simplified phantoms. Current *in silico* FEA-FIELD II approaches are computationally prohibitive for large-scale data generation and lack heterogeneous tissue support. This paper introduces Elastosynth, an open-source package with modular architecture: procedural phantom generation, a custom lightweight finite element solver, and optimized FIELD-II RF simulation. The generator employs Monte-Carlo placement and superpixel heterogeneity workflows for complex stiffness fields with tunable inclusion properties. The bespoke C++/Armadillo FE engine supports heterogeneous 2D simulations, bypassing commercial preprocessing overhead while efficiently computing displacements under prescribed boundary conditions. A lateral windowing scheme reduces the RF simulation time 60-fold, enabling mass generation of reasonably realistic *in silico* phantoms. Supporting automated batch processing, Elastosynth facilitates statistically significant evaluation of displacement estimation techniques, addressing a critical gap in elastography and enabling advancement of tissue motion tracking and quasi-static ultrasound elastography techniques.

## 1. Introduction

A major challenge in ultrasound elastography (USE) hampering the development of clinically viable technologies is the scarcity of high-quality radio-frequency (RF) data. This scarcity makes it difficult to distinguish algorithmic flaws from data deficiencies. Furthermore, performance evaluation is hindered by the lack of ground-truth clinical data and the oversimplified nature of physical tissue-mimicking phantoms (TMPs), which do not adequately reflect *in vivo* complexity.

Researchers have therefore turned to synthetic datasets generated by combining finite-element solvers (e.g., ABAQUS (ABAQUS 2019, Dassault Systèmes Simulia Corp., Johnston, RI, USA)) with ultrasound simulation tools. Among such tools, FIELD II [1,2] has been used extensively to generate reasonably accurate RF data for the development of machine learning based models of tissue displacement estimation [3,4,5,6,7,8]. This has made possible the creation of reasonably good-fidelity RF data with known ground-truth displacements for accuracy assessment. Other simulators offer different trade-offs: k-Wave [9] solves the full wave equation and is more rigorous but considerably slower, whereas SIMUS [10,11] and FOCUS [12] prioritize fast field computation, and convolution-model simulators [13,14,15,16] gain speed by convolving the scatterers with a fixed or depth-dependent point spread function, whereas Elastosynth keeps the FIELD II field computation for every RF line and only omits scatterers far from it. Elastography data have also been generated by coupling FIELD II with general-purpose FE packages such as FEMLAB 2.3 software (Comsol Inc. Burlington, MA, USA) [17] or FEBio [18]; neither study reports a restriction of the scatterers per RF line, continuously heterogeneous stiffness fields or seeded batches of distinct phantoms. These approaches also face two key limitations: FIELD II is still computationally intensive, restricting sample sizes, and commercial solvers lack user-friendly support for highly heterogeneous tissue models that mimic biological tissue, as such simulations remain a niche application.

Current development and testing procedures in USE remain limited, typically relying on manufactured TMPs or synthetic phantoms [19,20,21,22,23,24]. The TMPs are largely homogeneous with one or a few circular inclusions, presenting two major drawbacks: (1) challenging large-scale production required for machine learning applications, and (2) inability to replicate the continuous heterogeneity characteristic of biological tissues. Researchers therefore have often utilized *in silico* phantom generation via FIELD II. Yet the preference remains for locally homogeneous *in silico* phantoms, which largely stems from the lack of user-friendly means to develop highly heterogeneous finite-element (FE) models essential for estimating post-compression scatterer displacements. Major commercial FE solvers are primarily designed to accommodate uniform material sections, a common engineering use case in industrial applications, and poorly support continuously variable stiffness fields. Since biological tissues are inherently heterogeneous, this gap often makes algorithm validation challenging without ground-truth data. This motivates our first design objective: to provide an open-source user-friendly framework for specifying and simulating ultrasound interactions with arbitrarily complex material properties. Such a tool would enable reasonably high-fidelity synthetic data generation, facilitating rigorous evaluation of quasi-static USE techniques, including tissue motion tracking algorithms. Another issue encountered in simulated phantoms is the extensive computation time. This is due to superimposing the response of all scatterers within the phantom’s domain. However, it can be demonstrated that the actual signal captured by the ultrasound transducer is only influenced by the scatterers near the desired sampling points. As such, the second design objective is to identify the smallest lateral window of scatterers that leaves displacement estimation unaffected.

To address these gaps, we developed an open-source software package, henceforth referred to as Elastosynth (version 1.0.0.2), featuring an optimized FIELD II pipeline and a custom finite-element solver that natively supports heterogeneous 2D simulations. Our procedural generation algorithm produces random, reasonably realistic *in silico* phantoms with tunable properties and continuous tissue variability. By reducing the RF simulation time by up to two orders of magnitude, this method enables the mass generation of unique datasets, facilitating statistically significant and reproducible evaluation of displacement estimation and quasi-static USE algorithms.

## 2. Materials and Methods

Elastosynth is built around four key design objectives:

1. Provide a convenient image-based interface for specifying arbitrarily heterogeneous material property fields.
2. Enable high-throughput procedural simulation for generating large volumes of unique elastography samples.
3. Support an adaptable workflow ranging from fully automated batch generation to highly customizable simulations.
4. Accelerate data generation runtime through algorithmic optimization.

The overall functionality is organized into three major modules, whose roles and capabilities are summarized in Table 1.

### 2.1. In Silico Phantom Generation

**Figure 1:**
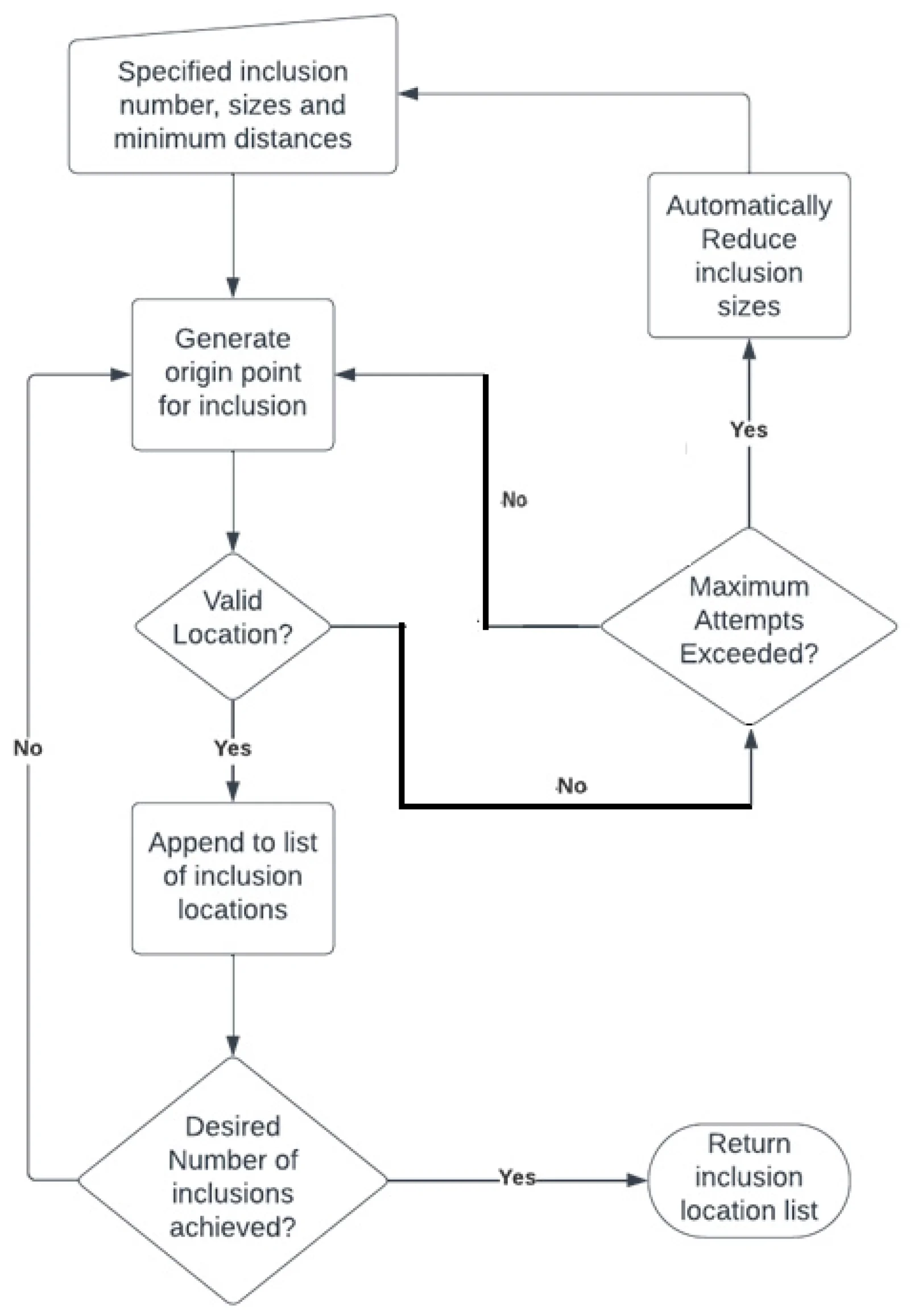
The algorithm used to generate randomized inclusion locations for the elastography phantom uses a Monte-Carlo approach to finding valid locations.

The procedural phantom generator creates unique phantoms for USE by randomly generating inclusions based on user-specified parameters—namely, their count, size, stiffness, and inclusion tissue heterogeneity. The first major methodological innovation is a Monte Carlo–based randomization [25] of inclusion locations, which ensures a valid spatial configuration, as depicted in Figure 1.

The algorithm takes as input a set of inclusion sizes, the minimum inter-inclusion distance, and the desired number of inclusions. It initializes the first inclusion at a random location. For each subsequent inclusion, random locations are generated and validated against the constraints (non-overlap and minimum distance) until either a valid location is found or the maximum attempt limit is reached. Once accepted, the location is appended to an array, and the process repeats until the target number of inclusions is generated. The attempt limit, a user parameter, was 10 per inclusion. If it is exceeded, the placement is restarted and all sizes are reduced by k + 5 pixels at the k-th restart, but not below 2 pixels. In dense phantoms this fallback shrinks the inclusions, and inclusions placed later tend to lie toward the periphery: with the requested radii of 40–50 pixels on a 256 × 256 grid used here, the fallback affected 32 of the 120 phantoms described in Section 2.2 and Section 2.4, including 17 of the 20 with four inclusions.

The module’s next major capability is a heterogeneity generator that transforms homogeneous inclusions and backgrounds into heterogeneous stiffness fields according to specified parameters. First, a “clean” stiffness image is created, featuring homogeneous inclusions and a background regions where each region can be assigned a Young’s modulus value based on respective mean values reported in the literature [26,27,28,29,30]. Second, Gaussian noise is added to introduce an initial layer of heterogeneity. Third, the noisy image is segmented into a variable number of regions via a super-pixel algorithm that groups pixels by proximity and similarity. These regions are then reassembled into the final image; within each, the Young’s modulus is adjusted by a randomly generated factor that alters the local Young’s modulus. This imparts a distinct Young’s modulus value to each super-pixel, yielding heterogeneous regions throughout the final image.

### 2.2. Finite Element Modelling

The FE engine of Elastosynth is a custom-designed C++ solver that leverages the Armadillo matrix library [31] and is tailored specifically for highly heterogeneous stiffness images. A tradeoff for this heterogeneity, however, is that the simulator is limited to 2D grid-based analyses to emulate USE. The solver implements bilinear quadrilateral elements in plane-stress and plane-strain formulations, selected per phantom. Moreover, because of its narrow scope and simple geometry, it can sidestep the extensive preprocessing and validation steps typical of traditional FE packages while remaining a lightweight solver module.

Both displacement components can be prescribed node by node on any of the four edges, including the lateral sides; edges without prescribed displacements are considered traction-free. Force boundary conditions are not supported, because the forces on the boundaries, in particular the lateral ones, cannot be measured reliably in quasi-static USE, whereas boundary displacements can be estimated from the RF data.

To evaluate the accuracy of the FE engine, we generated 100 phantom samples according to the method described in Section 2.1 (40 plane stress and 60 plane strain models, with 1–4 inclusions, a grid resolution of 256 × 256 elements, and a top displacement of −0.5 mm) and solved them with both the Elastosynth version 1.0.0.2 solver and ABAQUS/Standard 2025 (Dassault Systèmes, Abaqus Analysis User’s Guide, Version 2025. Providence, RI, USA: Dassault Systèmes Simulia Corp.) on the same mesh, with identical stiffness image, boundary conditions and Poisson’s ratio (0.45). For the displacement fields, accuracy was evaluated using the relative L_2_ error and the range-normalized root mean square error (NRMSE). The strain and stress fields were derived from their respective displacement solutions using a unified post-processing algorithm before comparison.

### 2.3. Radiofrequency Data Generation

FIELD II [1,2] handles the vast majority of the RF data generation. A general overview of the method is as follows:

1. Define the transducer and excitation parameters. This includes specifying the transducer geometry, the excitation pulse (center frequency, fractional bandwidth, sampling frequency), and the system impulse response.
2. Generate a scatterer field which involves creating a set of discrete scatterers with specific positions and assigning each a scattering amplitude.
3. Define the scan sequence and beamforming parameters. For each RF line, this involves specifying the transmit focus, transmit apodization, receive dynamic focusing range, receive apodization, and receive delays.
4. Loop over each transmit event (RF line), including the following for the current line:
  - Set the transmit aperture parameters (apodization and time delays) to steer/focus the beam.
  - Set the receive aperture parameters (apodization and dynamic focusing delays) for the current imaging depth.
  - Call FIELD II to compute the spatial impulse response from the transmit aperture to an optimal set of scatterers as per Section 2.4 and the spatial impulse response from the scatterers to the receive aperture. It also convolves these responses with the excitation pulse before summing the contributions from the scatterers to produce a single time-varying RF signal.
  - Store the resulting RF time-signal.
5. Repeat Step 4 for every RF line in the scan plane to assemble the complete RF dataset.

#### 2.3.1. Paired RF Data Generation for US Elastography

In the context of quasi-static USE, the core code process remains largely unaltered; however, to capture the deformation of the underlying tissues, two RF data frames are generated as described above. The first uses the original scatterer positions, while the second uses scatterers displaced according to the simulation performed by the FE engine. For each scatterer, the displacement is interpolated using the nodal displacements obtained from the FE analysis and the respective finite element’s shape functions. The new coordinates of each scatterer are calculated by adding its respective interpolated displacement to its coordinates in the reference undeformed state.

#### 2.3.2. Qualitative Assessment of Generated RF Data

For the qualitative assessment of the RF data generated using Elastosynth, Young’s modulus images reconstructed from the data were visually compared with those reconstructed from clinical patient RF data reported in [32,33]. The visual comparison focused on features such as tissue heterogeneity. The Young’s modulus images were generated using the reconstruction procedure described in [22], which involves processing the RF data with the AM2D [32] and STREAL [21] algorithms to compute tissue strain images, followed by an iterative algorithm [34,35,36] for the final Young’s modulus reconstruction.

### 2.4. Optimization Procedure

To address the issue of long computation times, an optimization procedure is introduced by which the scatterers involved in the calculation of the radiofrequency response of the tissues are truncated to only those within a certain distance of the query points. Normally, the entire response from all scatterers is calculated from the pressure wave emission, however the actual signal calculated in the post-beamformed data from Elastosynth is only influenced by the scatterers near the desired sampling points. Solving the full acoustic response over the entire spatial domain is therefore unnecessary, as each element in a linear US array probe receives negligible energy from most of the domain being stimulated except for a very narrow beam within the domain: the ultrasound transmit beam is narrow and the receive sensitivity is strongly directional. Furthermore, the two-way point spread function attenuates contributions from scatterers located at high angles from the beam axis.

A spatial window was defined for each RF line, passing only scatterers within a lateral distance of ±*w*·*L* from the line to FIELD II, where *L* is the total lateral width of the scatterer field. Consequently, each RF line utilizes approximately 2*w* of the scatterers. To evaluate the effect of *w*, partial- and full-domain simulations were compared across 20 phantoms at two levels: at the RF level, consistency was evaluated using normalized cross-correlation and envelope SNR; at the displacement level, the RMSE of the AM2D estimate [32] was computed against both the full-domain estimate and the FE ground truth.

### 2.5. Supported Workflows

**Figure 2:**
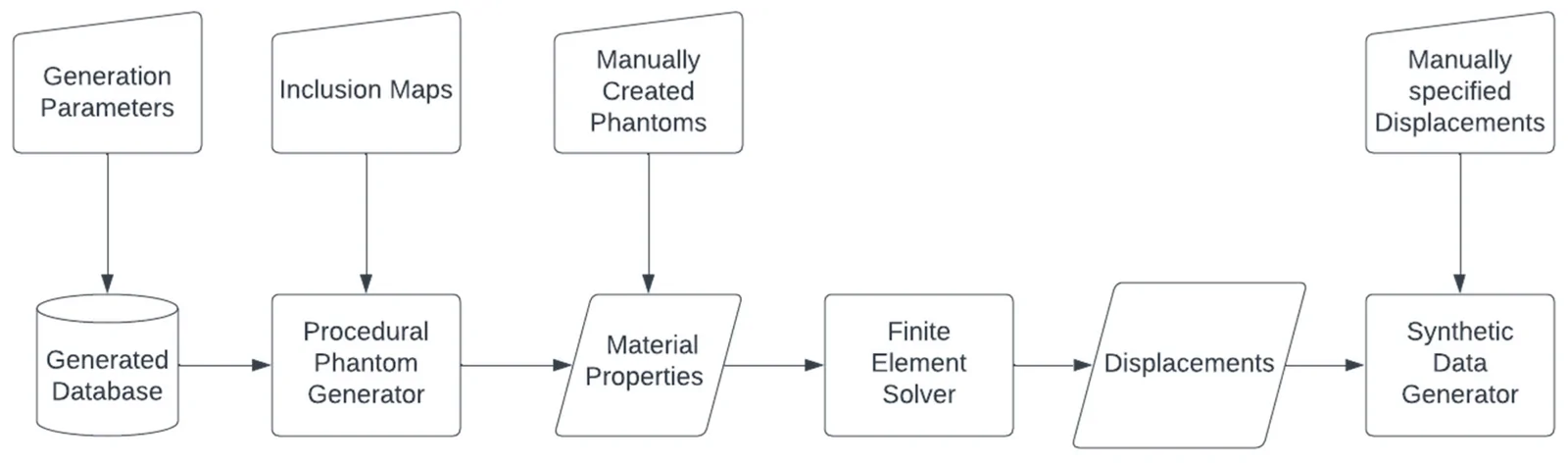
The currently supported workflows center on a main pipeline that initiates with a parameter dataset and progresses through the complete generation sequence. At any stage, however, users can input custom data to bypass earlier steps, thereby enabling manual and custom phantom generation.

Figure 2 presents a flowchart of the currently supported workflows. This list may not be exhaustive at the time of publication, as future updates may introduce additional features. The primary pathway begins with the generation of a parameter dataset, which contains the information required for creating the *in silico* phantoms. This dataset then feeds into a generator that constructs the underlying Young’s modulus distribution. Subsequently, data from the finite element solver is passed directly to the RF generator. At each stage, however, users can inject their own custom data, thereby bypassing any of the preceding steps.

The most general and automated workflow, which progresses from left to right, involves specifying a series of parameters to systematically generate any number of *in silico* phantoms. The next supported workflow enables manual specification of inclusion maps in conjunction with the built-in heterogeneity generator. If the material properties are known, they can be specified explicitly. Finally, if the desired displacements are known, they can be passed directly to the RF pair generator.

### 2.6. Hardware/Software Specification and Distribution

Elastosynth runs on a standard CPU and does not need a GPU; for a (256 × 256)-element phantom, each MATLAB (R2024a, The MathWorks Inc., Natick, MA, USA) worker along with its FE solver process, uses up to about 1.8 GB of RAM (about 4.3 GB of committed memory). On the workstation of Section 3.5, batches with 1, 2, 4 and 8 parallel workers produced 113, 219, 405 and 640 complete samples per hour (1% window); a sample saved by the batch scripts occupies about 7 MB.

Elastosynth is currently available as source code or as a MATLAB toolbox, precompiled for Windows and Linux. While compilation for macOS is expected to work, this has not been validated, nor is a precompiled version provided. Documentation is available at https://elastosynth.notion.site/43961d8dc2ea4bd29e8fc607856493c5 (accessed on 28 October 2026). The package is available on GitHub (https://github.com/CaiusMedical/Elastosynth, accessed on 28 October 2026) under the LGPL-2.1 license, and the version used in this paper (1.0.0.2) is archived on Zenodo (DOI 10.5281/zenodo.22901942). Phantom generation is seeded: a master seed assigns a seed to every phantom, so that any phantom can be regenerated from its row of the parameter table.

## 3. Results

### 3.1. Generated Tissue-Mimicking Phantoms

**Figure 3:**
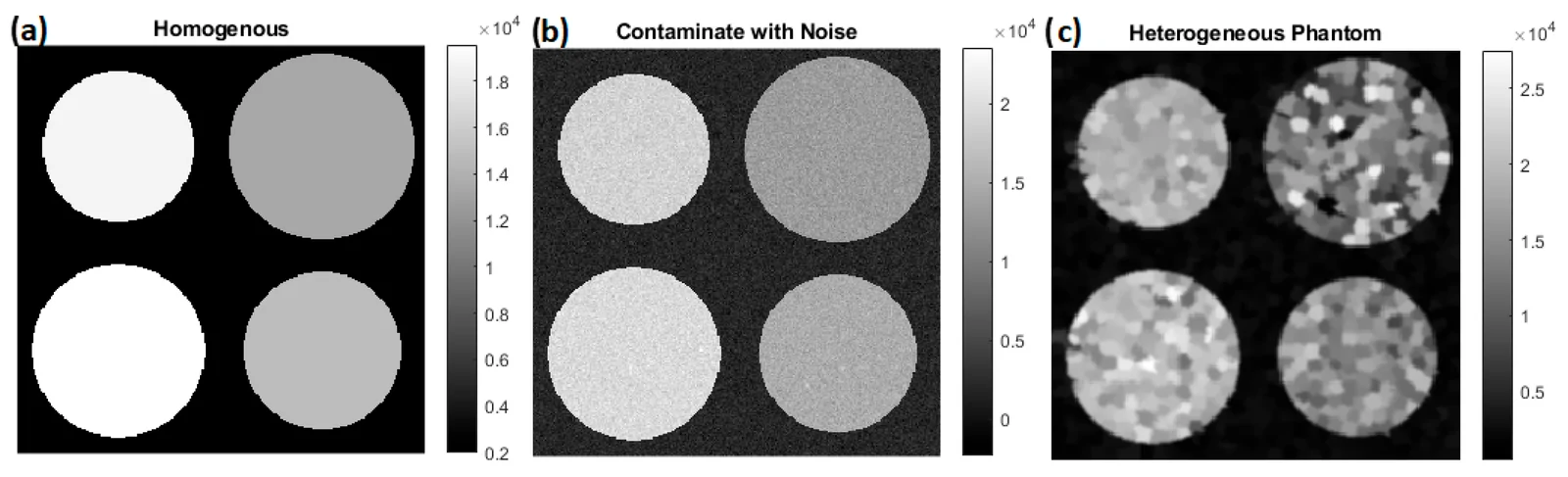
Generation of a heterogeneous stiffness field from an initially homogeneous image (**a**). This was achieved through a three-step process which begins with noise contamination of the homogeneous *in silico* phantom (**b**), followed by superpixel segmentation and then further changing the superpixel stiffness values which results in a heterogeneous phantom (**c**).

Figure 3 illustrates a sample of results obtained from the three-step process of heterogeneous *in silico* phantom generation. Consistent with the known heterogeneity of malignant tumors [37,38,39,40,41], this figure shows the generated ‘patchy’ nature of tumor tissue, with stiffer regions intermingled with softer areas.

### 3.2. Finite Element Modelling Results

**Figure 4:**
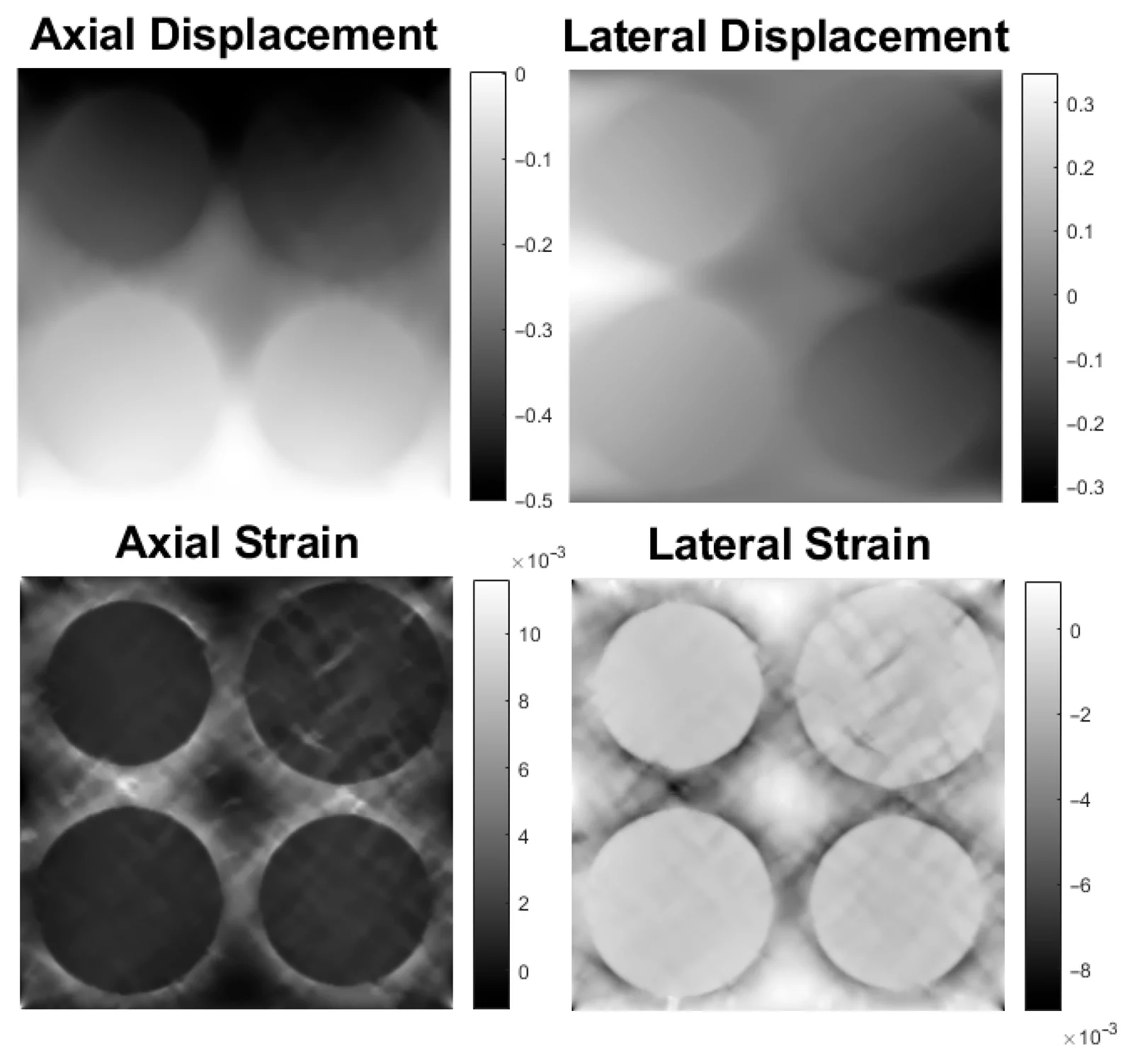
Finite element simulation of the phantom illustrated in Figure 3 where axial and lateral displacement and strain maps are illustrated.

Figure 4 illustrates sample outputs of displacement and strain fields from an *in silico* plane-stress phantom. In this phantom, a zero-displacement boundary condition was imposed at the bottom, while a prescribed displacement of −0.5 mm was applied at the top. As expected, the axial and lateral displacement images exhibit approximate symmetry and anti-symmetry, respectively, around the vertical midline. Moreover, both the axial and lateral strain images show approximate symmetry around the same line. Deviations from perfect symmetry and anti-symmetry are due to imperfect symmetry in the phantom’s geometry and stiffness distribution around the midline. The heterogeneity in both the inclusions and the background is clearly visible in the strain images.

**Figure 5:**
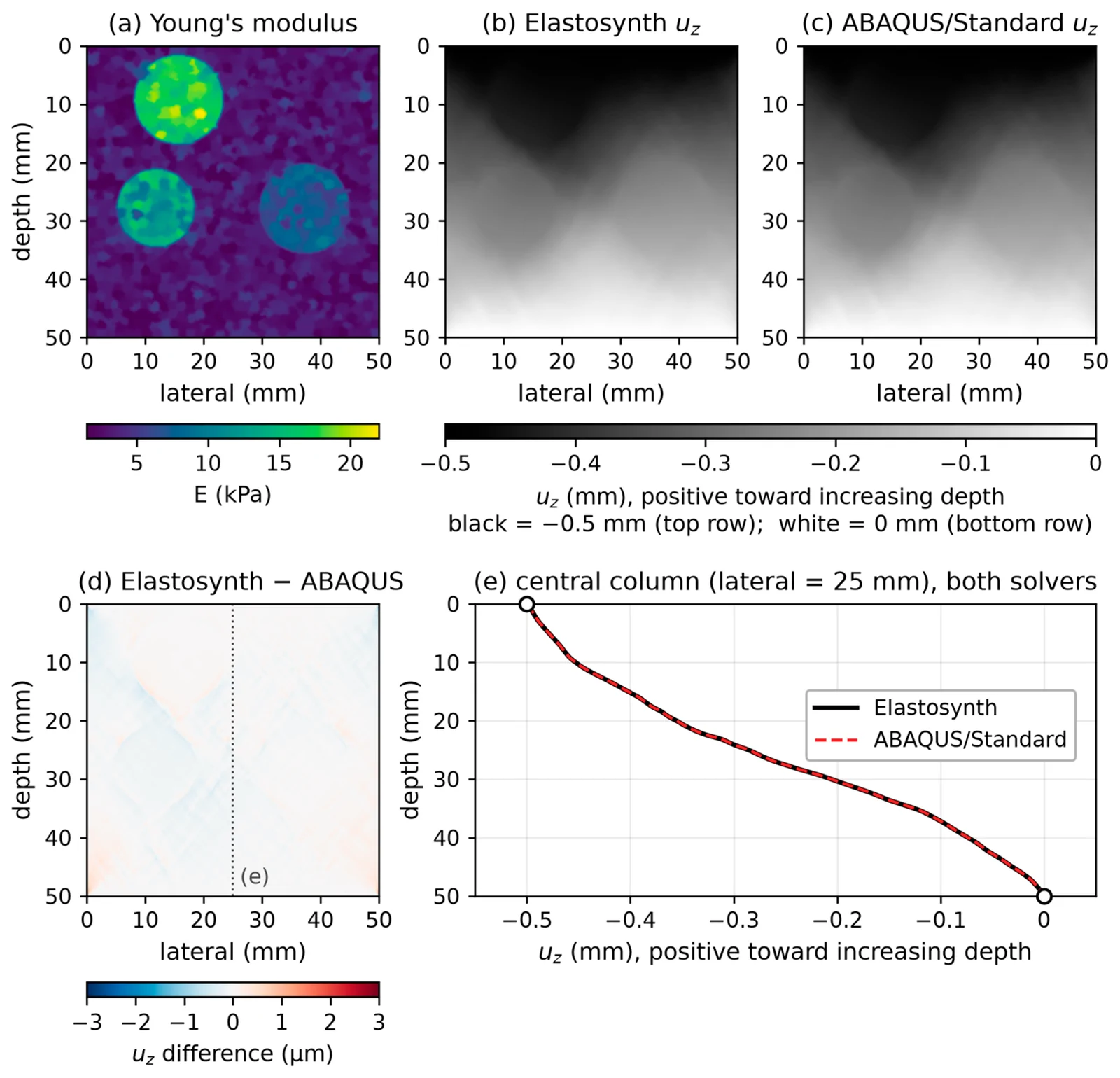
A plane-strain validation case: (**a**) Young’s modulus of the phantom; (**b**) axial displacement from the Elastosynth solver and (**c**) from ABAQUS/Standard for a prescribed top displacement of −0.5 mm with the bottom fixed; (**d**) difference between the two, with a maximum of 3 µm; (**e**) axial displacement along the central column.

**Figure 6:**
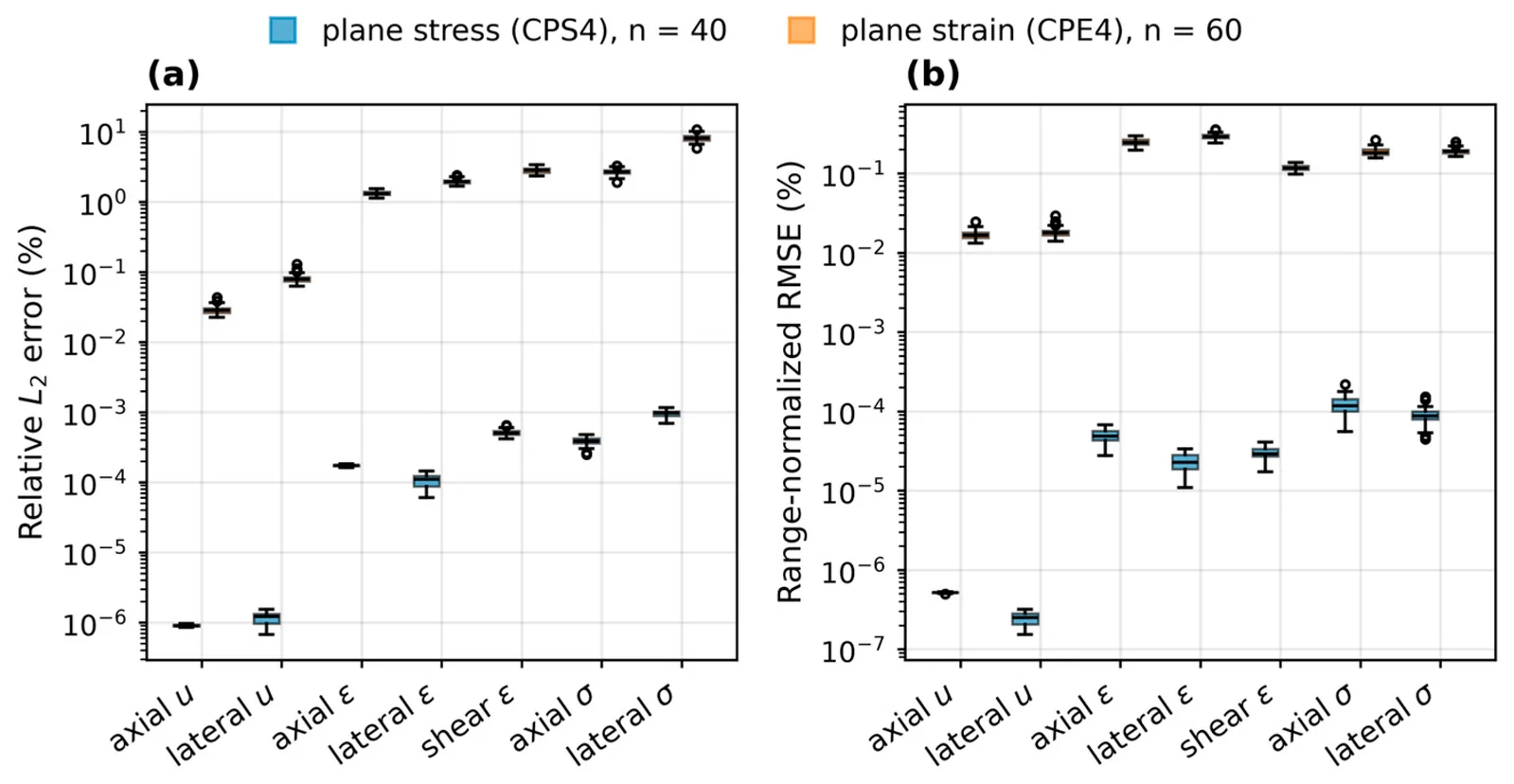
Case by case validation errors of the Elastosynth FE solver against ABAQUS across 100 phantoms (40 plane stress, 60 plane strain): (**a**) relative *L*_2_ errors for displacement, strain, and stress fields; (**b**) range-normalized RMSE.

Figure 5 presents one of the plane-strain validation cases, and Figure 6 details the case-by-case error distribution across all 100 simulated phantom samples. Under plane-stress conditions, the numerical solutions from Elastosynth and ABAQUS achieved full solver-precision alignment, yielding a median relative *L*_2_ error of 9 × 10^−7^% for axial displacement with a maximum of 1 × 10^−6^%. Under plane-strain conditions, the median relative *L*_2_ errors for axial and lateral displacements were 0.03% and 0.08%, with maximum errors reaching 0.04% and 0.13% respectively, while the range-normalized RMSE remained below 0.03%. For the strain and stress fields, the range-normalized RMSE stayed below 0.4%, whereas the median plane-strain relative L_2_ error was 1.3–2.9% for the strains and 8.1% for the lateral stress (Figure 6a). The two metrics differ because the range used to normalize the RMSE is dominated by peaks at the four corners, where the two solvers also differ the most. The observed plane-strain offset stems from the selectively reduced integration used by ABAQUS to prevent volumetric locking in first-order elements. As expected, this offset grows with Poisson’s ratio: for the phantom of Figure 5, the plane-strain relative L_2_ error of the axial strain was 0.09% at *ν* = 0 and increased to 0.7%, 1.4% and 4.2% at *ν* = 0.4, 0.45 and 0.49, whereas under plane stress all relative L_2_ errors remained below 0.002% for every value of Poisson’s ratio.

#### Scatterers Distribution in the Post-Deformation State

**Figure 7:**
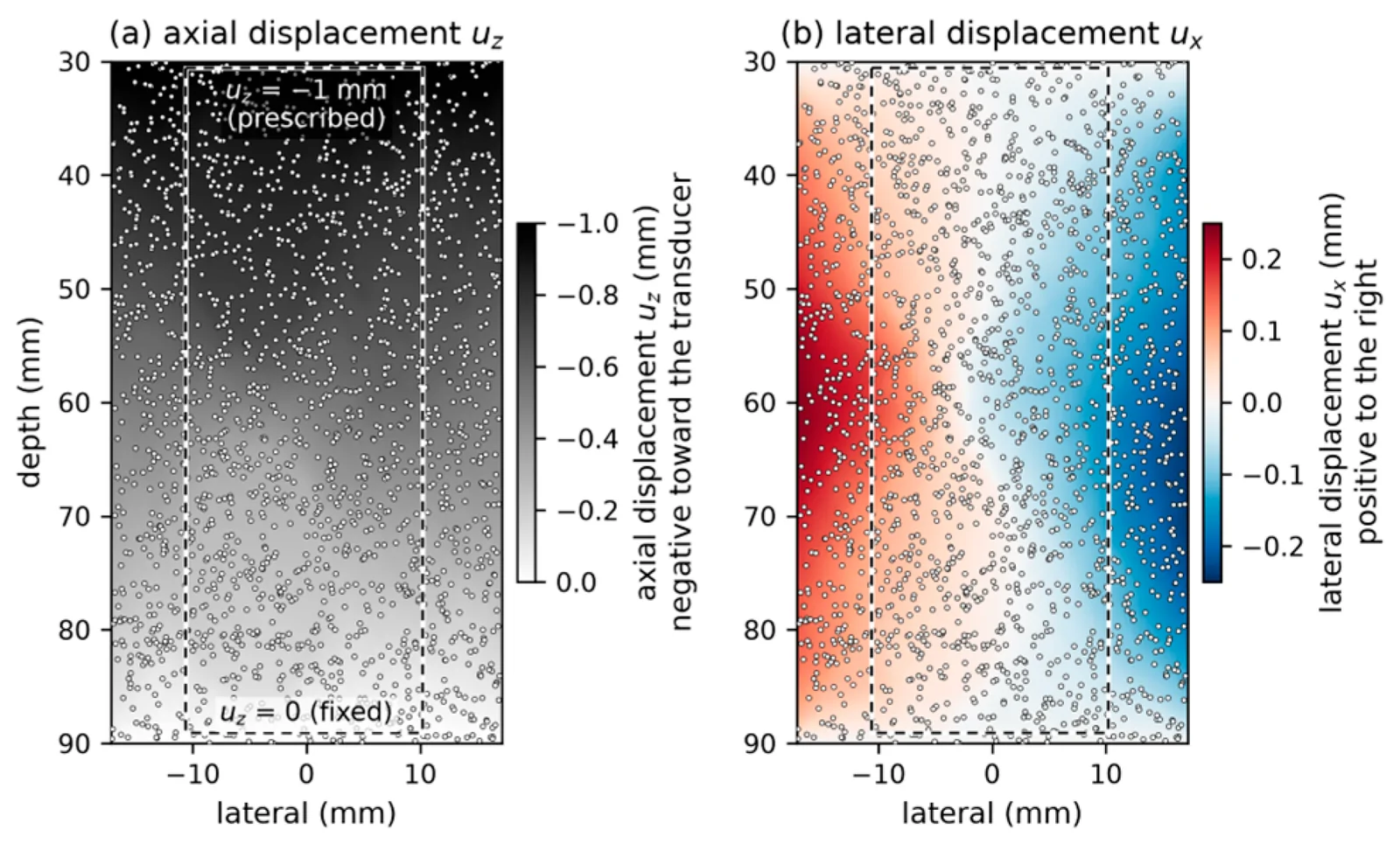
Axial (**a**) and lateral (**b**) displacements of the scatterers of one of the phantoms of Section 2.4. Depth is measured from the transducer; the scatterer field starts at 30 mm. Dots: a random 2% of the scatterers at their deformed positions; dashed box: region analyzed in Section 3.4.

Figure 7 presents the axial and lateral displacement fields for the randomly distributed scatterers in the *in silico* phantom, overlaid with the updated scatterer positions.

### 3.3. Assessment of Generated RF Data

**Figure 8:**
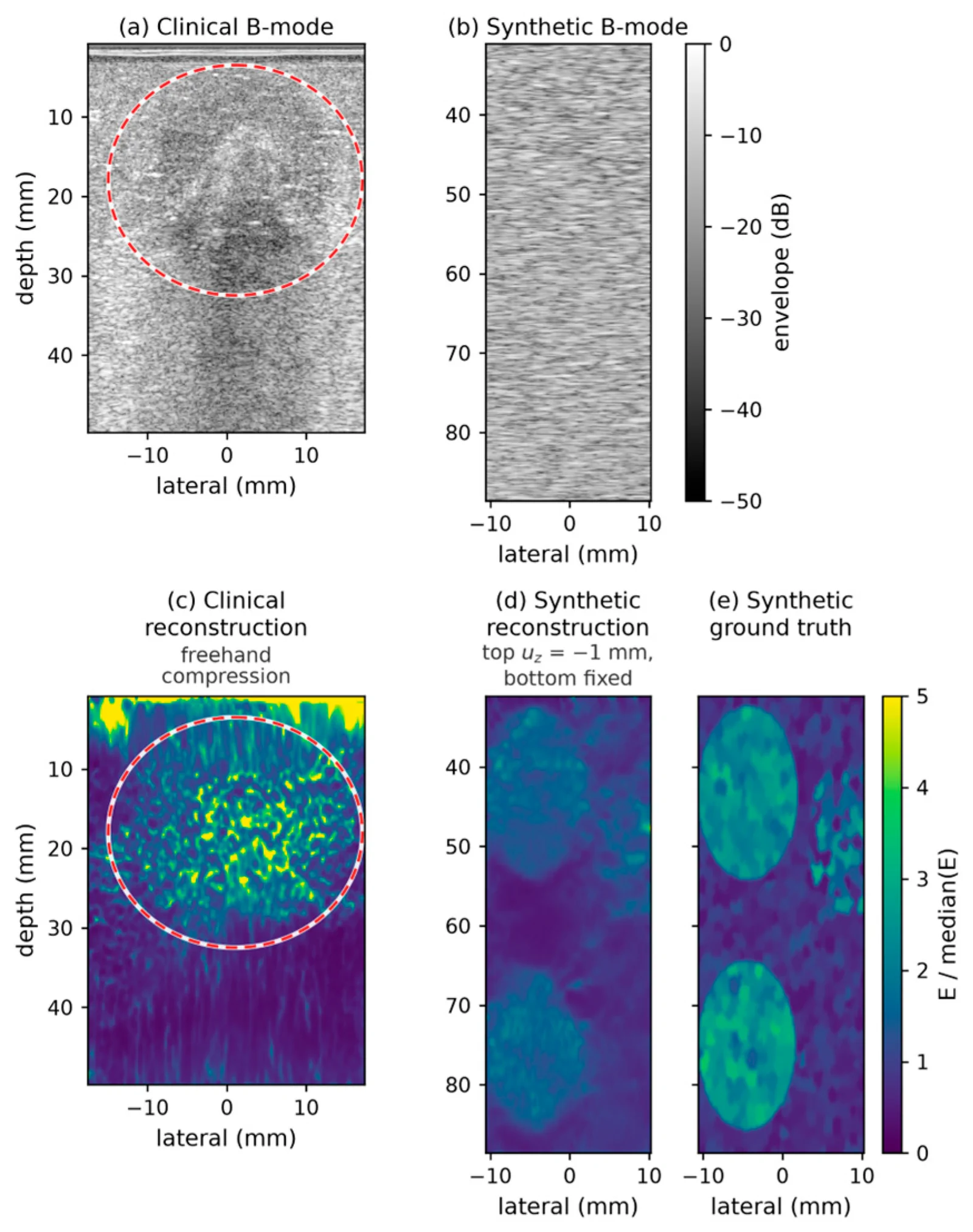
Qualitative comparison of a clinical case (**a**,**c**) and a synthetic case (**b**,**d**,**e**; 5% window): (**a**,**b**) B-mode images; (**c**,**d**) Young’s modulus images of the clinical and synthetic cases reconstructed with the same processing chain (STREAL step size reduced tenfold for stable convergence); (**e**) ground-truth modulus. Clinical: liver cancer patient [32,33] with tumor region of interest circled. Each modulus image is normalized by its median; all share one color scale. Inclusions are not visible in (**b**) because scatterer amplitudes in FIELD II do not depend on stiffness.

Figure 8 presents B-mode images and Young’s modulus images reconstructed from clinical RF data acquired from a liver cancer patient [32,33] and from synthetically simulated RF data generated using Elastosynth, both obtained with the same processing chain and algorithm settings. Both modulus images show stiffer, heterogeneous regions in a softer background; the comparison is qualitative, because tissue, transducer and loading differ between the two cases and the images depend on the reconstruction method. Overall, these results demonstrate that displacement estimators can be applied to synthetic data, with the key advantage that the results can be directly compared to the known ground truth.

A one-to-one comparison with clinical RF data is not possible, because the scatterer distribution and speed of sound in the patient are unknown; we therefore compared speckle statistics (medians over 3 mm × 6 mm tiles) between the clinical pre-compression frame of Figure 8 and synthetic frames simulated with a version of the RF module adapted to the clinical acquisition (6.67 MHz, 256 RF lines, dynamic receive focusing, attenuation 0.6 dB/cm/MHz). With 10^5^ scatterers, the speckle size matched the clinical frame (axial 0.25–0.26 vs. 0.25 mm; lateral 0.45–0.48 vs. 0.45 mm at the same tile positions); with 2 × 10^5^, the envelope SNR matched instead (1.59–1.61 vs. 1.61). The spectral centroid was lower (5.6 vs. 6.3 MHz), owing to the simulated attenuation and the unknown pulse and filtering of the scanner.

### 3.4. Optimization Procedure Assessment

**Figure 9:**
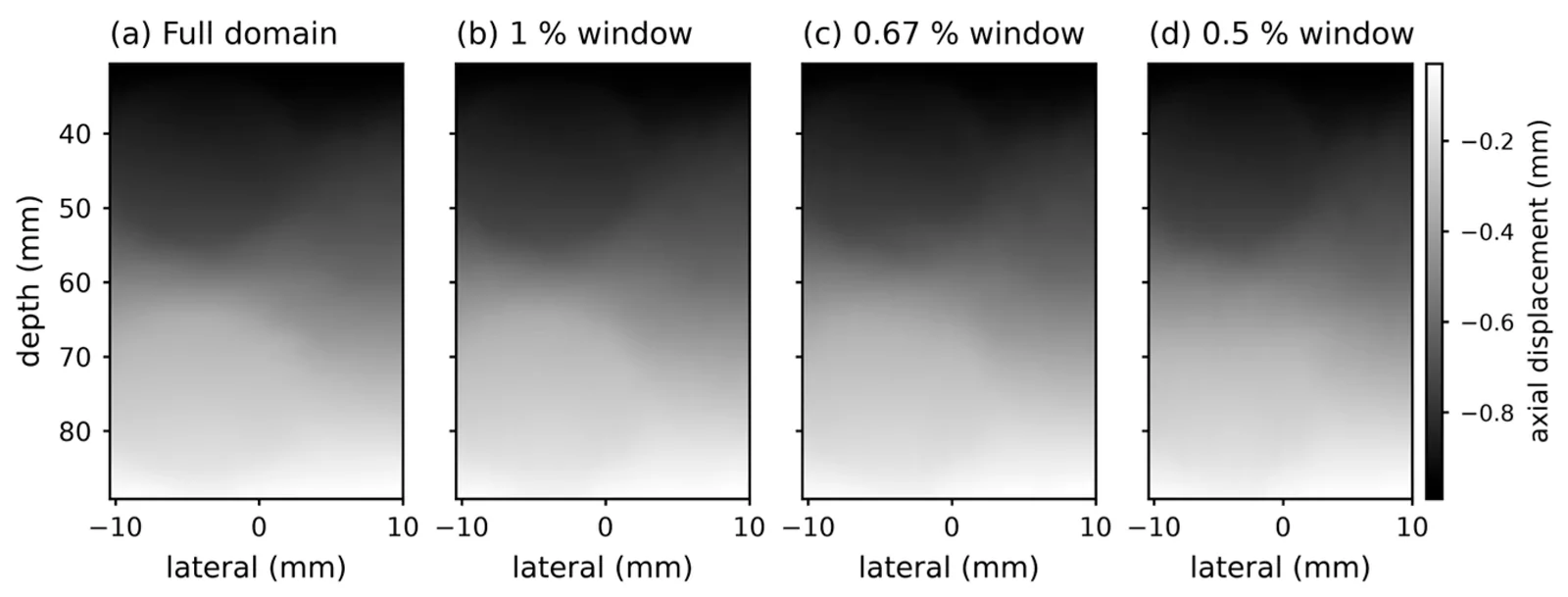
Axial displacement images estimated from RF data simulated with the full domain (**a**) and with lateral windows of 1% (**b**), 0.67% (**c**) and 0.5% (**d**).

**Figure 10:**
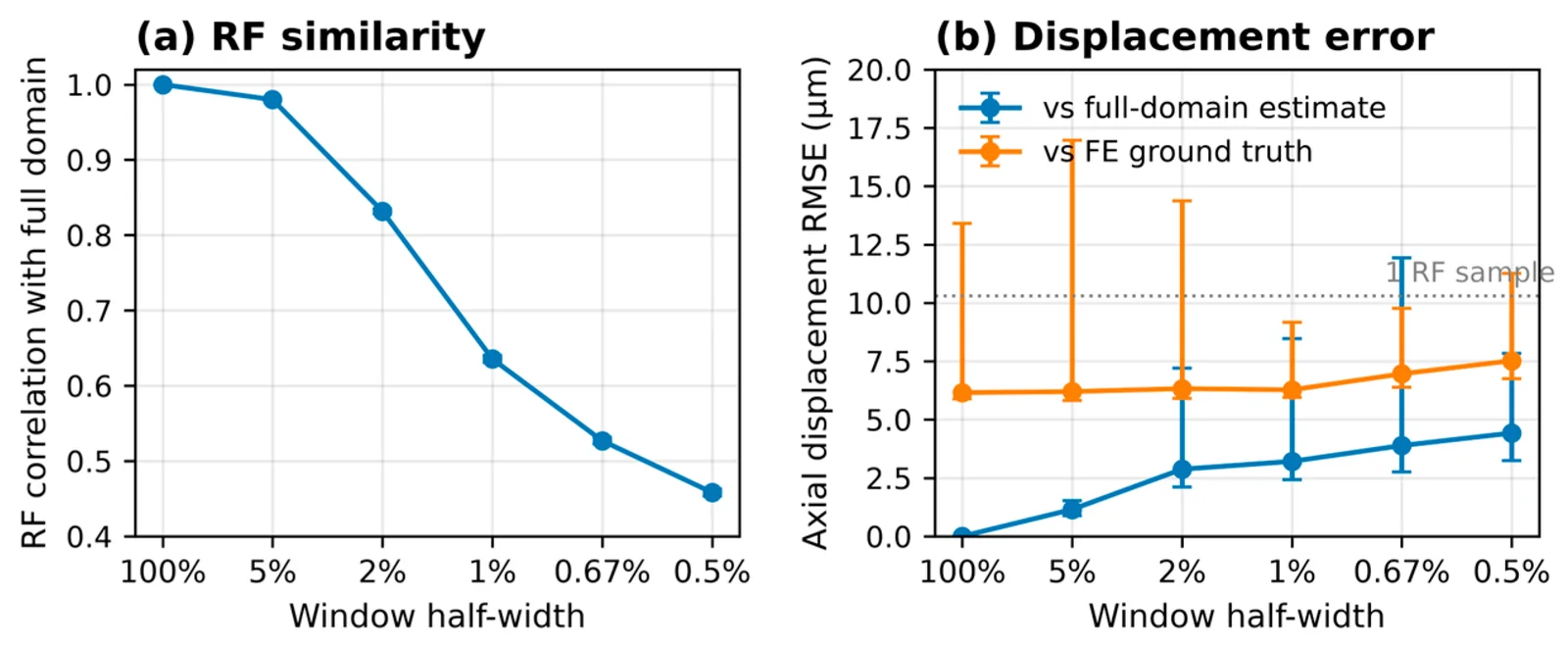
Impact of lateral window size across 20 phantoms. (**a**) Correlation coefficient between partial- and full-domain RF frames. (**b**) RMSE of AM2D axial displacement estimates relative to full-domain estimates (blue) and FE ground truth (orange).

Figure 9 illustrates the displacement maps for a representative phantom across the full domain and partial domains with window sizes of 1%, 0.67%, and 0.5%. The overall performance across 20 phantoms is summarized in Figure 10 and Table 2.

As the window narrows, the similarity between the partial-domain and full-domain RF frames declines (Figure 10a), with the correlation coefficient decreasing from 0.98 at a 5% window to 0.64 at a 1% window. This reduction is attributed to the window becoming narrower than the ultrasound beam profile in deeper regions. In contrast, displacement estimation accuracy remains virtually unaffected (Figure 10b): the discrepancy relative to the full-domain estimate increases to only 3.2 µm at a 1% window and 4.4 µm at 0.5%. Both values are lower than the error relative to the FE ground truth (6.2 µm for the full domain, 6.3 µm at 1% and 7.5 µm at 0.5%). The axial strain was calculated from these displacements using a least-squares estimator with a 1 mm kernel. Similar results were observed. Compared with the full-domain strain, the median relative L_2_ error was 11.5% at a 1% window and 4.4% at a 5% window. However, compared with the FE strain, the windowed strain showed no greater error than the full-domain strain.

Consequently, a 1% window achieves a 60-fold reduction in RF simulation time without sacrificing displacement estimation fidelity. For applications requiring faithful speckle statistics, a window size of 5% or wider is recommended.

### 3.5. Runtime Benchmark

All runtimes were measured on a single desktop workstation equipped with an AMD Ryzen 7 255 processor (8 cores, 3.8 GHz), 32 GB RAM, and Windows 11 Pro, running MATLAB R2024a, FIELD II 3.30, and ABAQUS/Standard 2025 in single-threaded mode. For a (256 × 256)-element phantom, the Elastosynth FE module required 11.2 ± 1.7 s, compared to 19.7 ± 0.3 s for the equivalent ABAQUS job. Note that every ABAQUS job includes startup, license checkout, and input preprocessing overhead. The FE module is open source and reads the stiffness image directly, whereas the speed-up of the complete pipeline is primarily derived from the reduced model of lateral windowing. The RF simulation of one frame pair took 1451 ± 45 s with the full domain and 24 ± 3 s with the 1% window (Table 2), so that one complete sample is generated in about 35 s instead of about 25 min.

## 4. Discussion

This Elastosynth software package successfully achieves its goal of providing a user-friendly package for generating synthetic quasi-static USE data using FIELD II as the ultrasound simulation tool combined with an in-house FE solver. We implemented and validated a highly efficient procedural RF data generation tool capable of producing data for randomly distributed and heterogeneous inclusions within a specified field of view. Additionally, we incorporated the FE solver, rigorously validated against ABAQUS, to handle continuously variable stiffness distributions, albeit currently limited to a square field of view. Furthermore, the partial-domain approach accelerates the RF simulation 60-fold with a 1% window and 101-fold with a 0.5% window, enabling the generation of massive datasets necessary for developing deep learning-based models of USE such as [3,4,33,42,43].

By substantially expanding the capabilities of *in silico* synthetic data generation, our approach enables the creation of arbitrarily heterogeneous datasets, allowing for statistically significant comparisons of algorithm performance. Moreover, these new capabilities allow generation of reasonably realistic clinical examples, including tumors that are well-integrated with the background tissue without distinct boundaries. This feature allows capturing tumor characteristics specific to various cancer types such as breast cancer where the “stiff rim” phenomenon, which pertains to elevated stiffness of the outer shell of malignant breast tumors, is well documented [44,45,46,47].

The FE solver is 2D because quasi-static USE is commonly performed on 2D image planes under plane-stress or plane-strain assumptions. As FIELD II is already 3D, scatterer displacements from an external 3D solver can be passed to the RF generator; a 3D solver will be considered as 3D USE becomes more common.

Future work includes expanding the FE solver to accommodate variable inclusion morphology, including shape and multi-focal characteristics. It also includes expanding the FE solver to accommodate additional element types beyond plane strain and plane stress, leveraging real clinical data to train high-fidelity generative AI models, and incorporating the capability to simulate purposeful data corruption. Another future direction includes using the k-Wave ultrasound simulator [9], which, while more computationally expensive, is known to be more rigorous than FIELD II.

## Figures and Tables

**Table 1:** Elastosynth comprises three major modules, each fulfilling a specific task and capable of independent operation. The procedural phantom generator constructs phantom geometries based on user-defined parameters. The second module performs FE modeling to simulate phantom compression. The final module generates radiofrequency data using FIELD II.

Procedural Phantom Generation Module	Finite Element (FE) Modelling Module	Radiofrequency Data Generation Module
Generates unique *in silico* phantoms based on predetermined parameters, including inclusion count, size, location, stiffness, and tissue heterogeneity.	Simulates a given *in silico* phantom under user-defined boundary conditions, producing a displacement field for subsequent ultrasound data simulation.	Generates RF data via FIELD II, using the simulated displacement field obtained from the FE module along with user-defined transducer and imaging parameters.

**Table 2:** Comparison of partial- and full-domain simulations across 20 phantoms (plane stress, Poisson’s ratio 0.45, top displacement −1 mm, 10^5^ scatterers, the package’s L12-3V transducer definition: 7.5 MHz, 72 RF lines). Window: half-width as a fraction of the lateral extent. NCC and envelope SNR (over the whole analysis region, without depth-gain compensation) are means, and RMSE values are medians, over 20 phantoms; one RF sample is 10.3 µm. Times are per frame pair, mean ± SD of three phantoms run serially.

Window	RF NCC	Envelope SNR	Axial RMSE vs. Full (μm)	Frame Pair Time (s)	Speed Up
100%	1.000	1.50	0	1451 ± 45	1
5%	0.980	1.45	1.1	133 ± 35	11
2%	0.832	1.26	2.9	44 ± 1	33
1%	0.636	1.09	3.2	24 ± 3	60
0.67%	0.527	0.99	3.9	18 ± 4	81
0.5%	0.458	0.91	4.4	14 ± 3	101

## Data Availability

The radio-frequency data of the patient investigated in this article were kindly made available by Dr. H. Rivaz’s research laboratory.
